# Improving the electrocatalytic properties of Pd-based catalyst for direct alcohol fuel cells: effect of solid solution

**DOI:** 10.1038/s41598-017-05323-y

**Published:** 2017-07-07

**Authors:** Cuilian Wen, Ying Wei, Dian Tang, Baisheng Sa, Teng Zhang, Changxin Chen

**Affiliations:** 1 College of Materials Science and Engineering, Fuzhou University, Fuzhou, Fujian 350116 P.R. China; 2 National Key Laboratory of Science and Technology on Micro/Nano Fabrication, Key Laboratory for Thin Film and Microfabrication of the Ministry of Education, Department of Micro/Nano Electronics, School of Electronic Information and Electrical Engineering, Shanghai Jiao Tong University, Shanghai, 200240 P.R. China

## Abstract

The tolerance of the electrode against the CO species absorbed upon the surface presents the biggest dilemma of the alcohol fuel cells. Here we report for the first time that the inclusion of (Zr, Ce)O_2_ solid solution as the supporting material can significantly improve the anti-CO-poisoning as well as the activity of Pd/C catalyst for ethylene glycol electro-oxidation in KOH medium. In particular, the physical origin of the improved electrocatalytic properties has been unraveled by first principle calculations. The 3D stereoscopic Pd cluster on the surface of (Zr, Ce)O_2_ solid solution leads to weaker Pd-C bonding and smaller CO desorption driving force. These results support that the Pd/ZrO_2_-CeO_2_/C composite catalyst could be used as a promising effective candidate for direct alcohol fuel cells application.

## Introduction

Direct alcohol fuel cells (DAFCs) have been attracted great attention on their potential application in the portable electronics and automobile industries^1^. Among various liquid fuels, the methanol is volatile and relatively toxic, and ethanol has been recognized as the most suitable fuel as it is a sustainable and carbon-neutral transportation fuel^2^. However, it is difficult to break the C-C bond in ethanol at temperature lower than 100 °C, and the main product of ethanol oxidation reaction is acetate. As such, the electron transfer rate of ethanol in the alkaline DAFCs is only 33%^3, 4^. Ethylene glycol (EG) is a good choice to replace ethanol because the main product of the EG oxidation is oxalate^5, 6^, such that the ETR reaches 80%, which is much higher than that of ethanol in the alkaline DAFCs^4^. Also, EG has superior energy density (7.56 kWh L^−1^), higher boiling point (198 °C) and could be produced from biomass in great quantities^7–9^, which makes it as an appealing candidate for fuel cells application.

Pt-based catalysts have been investigated extensively as good electrocatalysts for low temperature fuel cells, especially for its acidic-resistant property^10^. However, compared to Pt electrocatalyst, Pd is more abundant, less expensive, and could promote EG oxidation more efficiently in alkaline media^11, 12^. A lot of efforts have been done to prepare the Pd-base catalysts with high catalytic activity and anti-CO-poisoning for alcohol electro-oxidation. In addition to the alloying approach^11, 13–16^, oxide-modified Pd has been reported to be superior to Pd/C catalyst for alcohol oxidation^17–22^. For instance, Li *et al*. reported that the addition MgO into Pd/C catalysts could significantly improve the reaction activity and the poisoning resistance for ethanol electro-oxidation due to the synergistic effect between Pd and MgO^19^.

CeO_2_ is as a well-known promoter in noble metal-based combustion catalysts. In addition, ZrO_2_ is always used as the support of Pt for water-gas shift in low temperature^23–25^. In recent studies, CeO_2_^26, 27^ and ZrO_2_^28–30^ have attracted increasing interests as the support of Pt or Pd and showed excellent catalytic activity. The Ce_x_Zr_1−x_O_2_ system has also been investigated because that the thermal stability, redox property and oxygen storage capacity could be improved by the addition of ZrO_2_ to CeO_2_^31–35^. However, there is few work reported about ZrO_2_-CeO_2_ mixed oxides as support materials on the EG electrocatalytic performance of Pd/C catalysts in the literatures. This prompts us to carry out an extensive and systematic investigation on this topic, which is of great interest and importance.

In this study, Pd/C, Pd/ZrO_2_/C, Pd/CeO_2_/C and Pd/ZrO_2_-CeO_2_/C composite catalysts have been prepared and characterized. The effect of the solid solution of ZrO_2_-CeO_2_ binary oxides as support materials on the electrocatalytic performance of Pd-based catalysts has been inverstigated. The physical origin of the good anti-CO-poisoning property of Pd_4_/ZrO_2_-CeO_2_ (111) composite catalyst has been unraveled based on first principle calculations as well.

## Results

### Structural characterization

**Figure 1:**
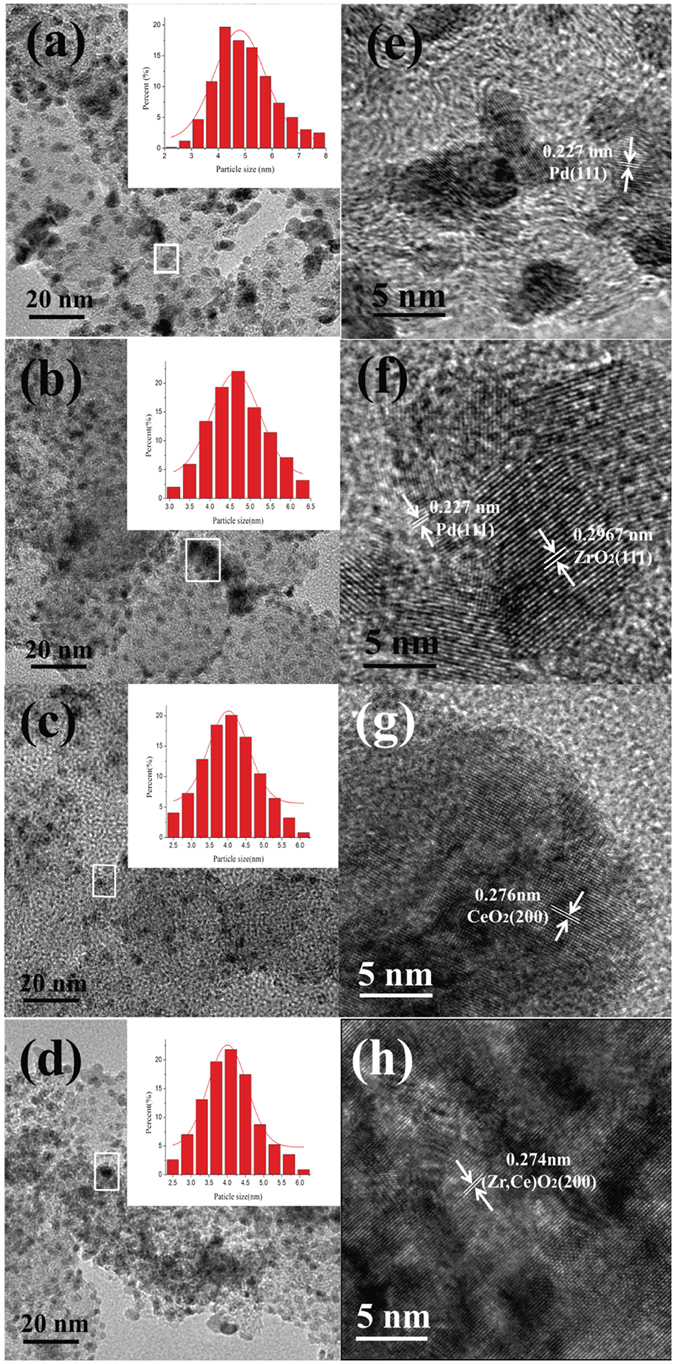
TEM images of different catalysts, for (**a**) Pd/C, (**b**) Pd/ZrO_2_/C, (**c**) Pd/CeO_2_/C and (**d**) Pd/ZrO_2_-CeO_2_/C. Insets show the corresponding size distribution in (**a**,**b**,**c**) and (**d**), respectively. (**e**,**f**,**g**) and (**h**) are the HRTEM images for (**a**,**b**,**c**) and (**d**), respectively.

The TEM images of different catalysts are shown in Fig. 1a–d. It is clearly that Pd nanoparticles of all catalysts are uniform and monodisperse. The average size of Pd is 4.8 nm for Pd/C, 4.1 nm for Pd/CeO_2_/C, 4.6 nm for Pd/ZrO_2_/C, and 4.0 nm for Pd/ZrO_2_-CeO_2_/C, respectively, as shown in Fig. 1a–d, consistent with that calculated on XRD data (in Fig. S1). It is noticed that the particle size of Pd changes more significantly with the addition of CeO_2_ than ZrO_2_ in this work. Similar results have been reported in Pt catalysts modified by Ce_0.6_Zr_0.4_O_2_^31, 32^.

It is observed that some grains with a d-spacing of 0.227 nm from the HRTEM images of all catalysts in Fig. 1e–h, which is assigned for the (111) plane of cubic Pd. And the d-spacing of some grains is 0.276 nm in Pd/CeO_2_/C (Fig. 1g), corresponding for the (200) plane of CeO_2_. While the d-spacing of some grains in Pd/ZrO_2_-CeO_2_/C (Fig. 1h) turns out to be 0.274 nm, which is smaller than that of Pd/CeO_2_/C. This could be related to the replacement of Ce^4+^ ions by Zr^4+^ ions, which results in a shrinking of the lattice volume of the solid. Based on the HRTEM results, one can confirm that the addition of 50% ZrO_2_ contributes to the formation of (Zr, Ce)O_2_ solid solution in present work.

**Figure 2:**
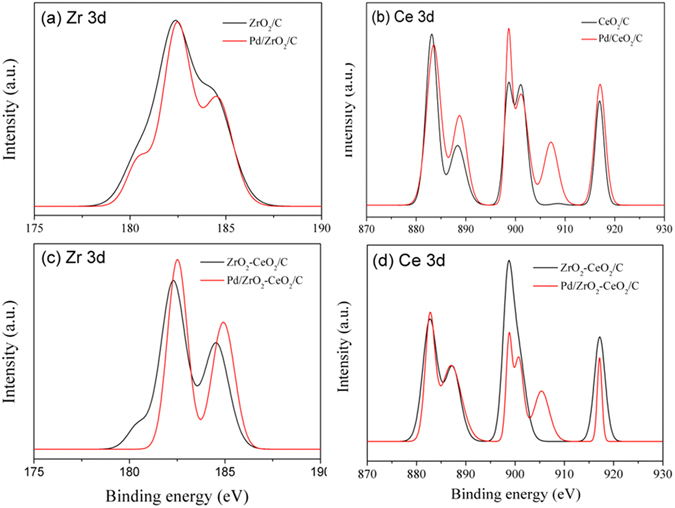
XPS spectra of Zr 3d and Ce 3d in different species before and after Pd-loading, (**a**) Zr 3d for ZrO_2_/C and Pd/ZrO_2_/C, (**b**) Ce 3d for CeO_2_/C and Pd/CeO_2_/C, (**c**) Zr 3d and (**d**) Ce 3d for ZrO_2_-CeO_2_/C and Pd/ZrO_2_-CeO_2_/C, respectively.

XPS was employed to investigate the nature of surface species for the catalysts. Figure 2 shows the XPS spectra of Zr 3d and Ce 3d in different species before and after Pd-loading. It is evident that the binding energy of Zr 3d_5/2_ slightly increases from 182.3 eV for ZrO_2_/C to 182.5 eV for Pd/ZrO_2_/C (Fig. 2a); while the binding energy of Ce 3d_5/2_ changes from 883.1 eV for CeO_2_/C to 883.6 eV for Pd/CeO_2_/C (Fig. 2b), which reveals the effect of Pd-loading on the electronic structure of Zr 3d or Ce 3d. Similar change in binding energy has been observed in Zr 3d_5/2_ (Zr 3d_3/2_) as well as Ce 3d_5/2_ for ZrO_2_-CeO_2_/C after Pd-loading, as shown in Fig. 2c–d. The presence of ZrO_2_-CeO_2_ in the vicinity of Pd nanoparticles could be beneficial for the EG and CO oxidation^36^.

Moreover, the interaction between Zr and Ce was determined by the XPS analysis. It is found the Zr 3d_5/2_ peak of ZrO_2_-CeO_2_/C (Fig. 2c) shifts negatively about 0.1 eV compared to that of ZrO_2_/C (Fig. 2a), and the Ce 3d_5/2_ peak of ZrO_2_-CeO_2_/C (Fig. 2d) shifts negatively about 0.1 eV compared to that of CeO_2_/C (Fig. 2b). It indicates that there was an electronic interaction between the ZrO_2_ and CeO_2_, which could play an important role in catalytic reaction and the activation of both dispersed metal and oxide matrix during electrode process^37^. The electron cloud density of Pd could be changed by ZrO_2_-CeO_2_/C supporting, and a synergetic effect could be yielded to improve the catalytic activity of Pd catalyst.

**Figure 3:**
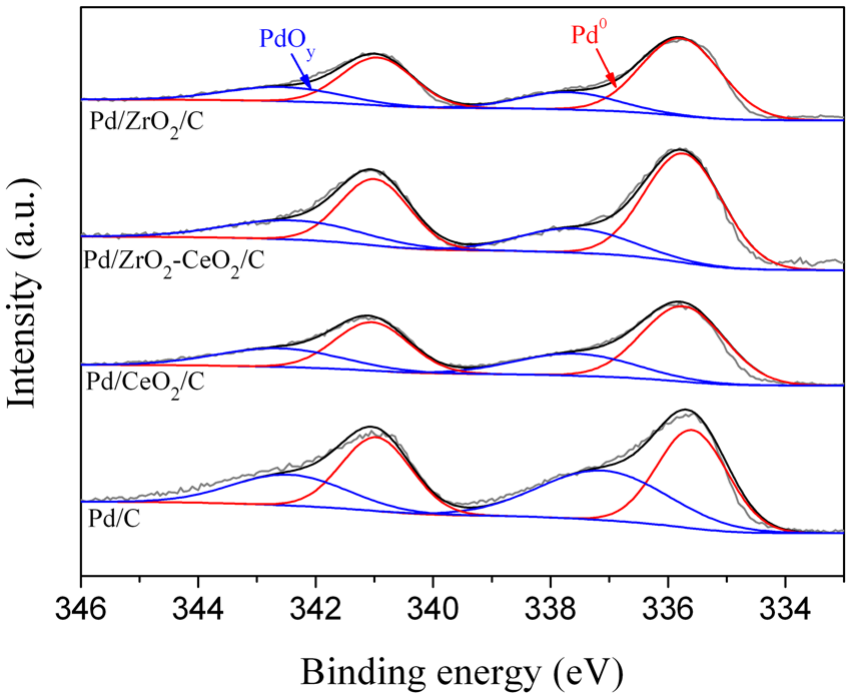
XPS spectra of Pd 3d for different catalysts.

The XPS spectra of Pd 3d in different catalysts are shown in Fig. 3. It shows that the peaks corresponding for Pd 3d_5/2_ (~335.7 eV) and Pd 3d_3/2_ (~341.0 eV) core levels can be ascribed to Pd and PdO_y_ (0 < y < 2) species, respectively^38, 39^. It is obviously that both Pd metal and PdO_y_ can be observed on the surface of prepared catalysts. The ratio of Pd: PdO_y_ is respectively calculated to be 1.2, 1.4, 2.0 and 2.3 for Pd/C, Pd/CeO_2_/C, Pd/ZrO_2_/C and Pd/ZrO_2_-CeO_2_/C. As reported in the literature^33^, CeO_2_ can act as an oxygen storage reservoir, which gives lattice oxygen to Pd and stabilizes Pd in an oxidized form. Therefore, the increased metallic Pd content can be mainly related to (Zr, Ce)O_2_ solid solution.

In addition, the binding energy of Pd 3d_5/2_ shifts positively from 335.65 eV for Pd/C to 335.70 eV for Pd/CeO_2_/C, and to 335.74 eV for Pd/ZrO_2_-CeO_2_/C. Combining with the XPS results in Zr 3d, Ce 3d and Pd 3d (in Figs 2 and 3), it can conclude that strong interaction exists between Pd and oxides, especially for the addition of (Zr, Ce)O_2_ solid solution. Moreover, the atom ratio of Pd: Zr: Ce is measured about 6.49: 0.98: 1.02 for Pd/ZrO_2_-CeO_2_/C catalyst, while the values are about 6.51: 1.92: 0 and 6.45: 0: 2.01 for Pd/ZrO_2_/C and Pd/CeO_2_/C respectively, indicating that Pd has been sucessfully loaded in the oxide support and the atom ratio of Zr: Ce is nearly 1: 1 for ZrO_2_-CeO_2_ solid solution.

### Electrochemical activity and stability

**Figure 4:**
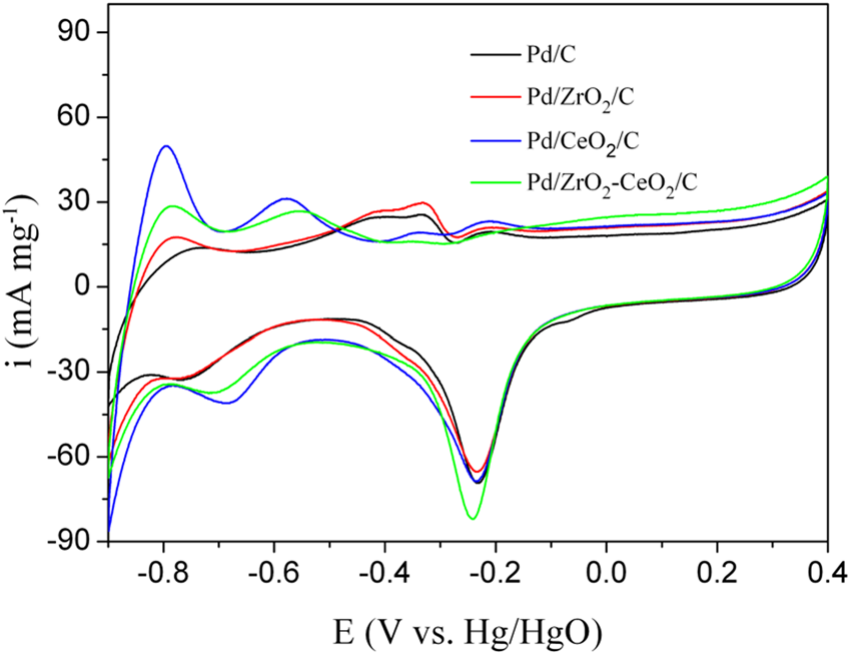
Cyclic voltammograms of different catalysts in 1 M KOH solution at a scan rate of 50 mVs^−1^.

Figure 4 shows the cyclic voltammograms of different catalysts in 1 M KOH solution. It is known that the position and size of anodic peak often change with the modification of Pd catalyst. In general, the anodic peak in the potential range between −0.9 and −0.7 V corresponds for the oxidation of the absorbed and adsorbed hydrogen, the peak from −0.6 to −0.4 V is for OH^−^ adsorption, and the peak at about −0.2 V can be ascribed to Pd oxide formation, respectively. In addition, the cathodic peak ranging from 0.0 to −0.4 V is due to the reduction of Pd oxide, and the peak between -0.6 to −0.8 V relates to the hydrogen adsorption/absorption^40, 41^. The reduction peak current density of PdO is 82 mA mg^−1^ for Pd/ZrO_2_-CeO_2_/C composite catalyst, which is significantly larger than other catalysts. This indicates the highest electrochemical activity of Pd/ZrO_2_-CeO_2_/C composite catalyst in present work.

**Figure 5:**
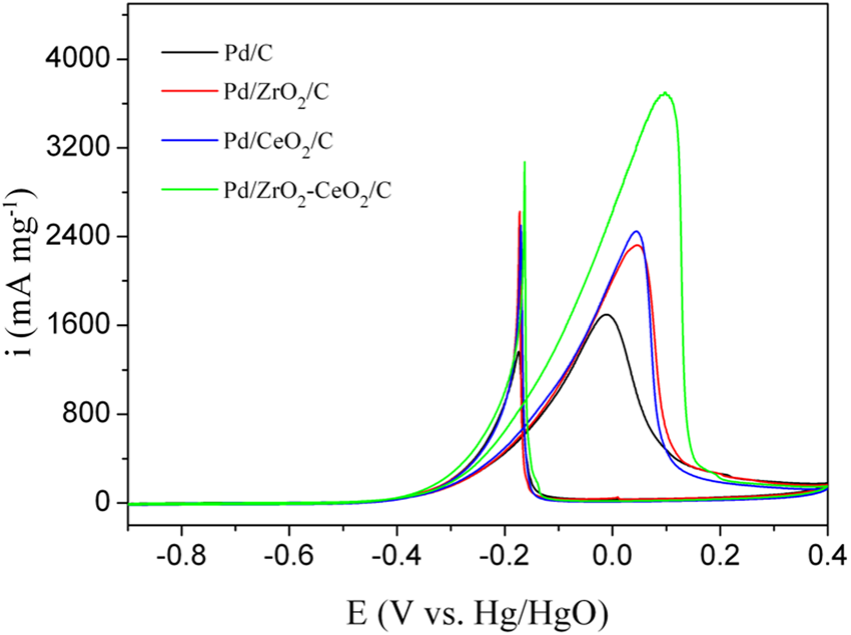
Cyclic voltammograms of different catalysts in 1 M KOH solution containing 1 M EG at a scan rate of 50 mV s^−1^.

Figure 5 shows the cyclic voltammograms of different catalysts in 1 M KOH solution containing 1 M EG. There are two anodic peaks observed in the forward and reverse scans. The oxidation current increases with the potential increasing for EG oxidation in the forward scan, then decreases due to the reduced activity of Pd and CO poisoning^43^. The anodic peak appeared during the reverse scan could correspond to re-oxidation process of the intermediate product of EG^17, 42^. It is noticed that the current density (i_f_) of forward anodic peak of Pd/ZrO_2_-CeO_2_/C (3700 mA mg^−1^) is much greater than that of Pd/C (1695 mA mg^−1^) and Pd/CeO_2_/C (2448 mA mg^−1^). This implies that the Pd/ZrO_2_-CeO_2_/C composite catalyst has highest catalytic activity for EG electro-oxidation. It has been reported that the positive shifts of Pd 3d_5/2_ peak of Pd-based catalysts indicates the decrease in the 3d electron density of Pd. The Pd with low 3d electron density is not easy to bind with the intermediate, and thus the intermediate coverage on Pd surface can be reduced^43^. Therefore, the highest catalytic activity of Pd/ZrO_2_-CeO_2_/C composite catalyst in present work can be attributed to the interaction between Pd and (Zr, Ce)O_2_ solid solution (in Figs. 2 and 3) as well as the smallest particle size of Pd (Fig. 1).

**Figure 6:**
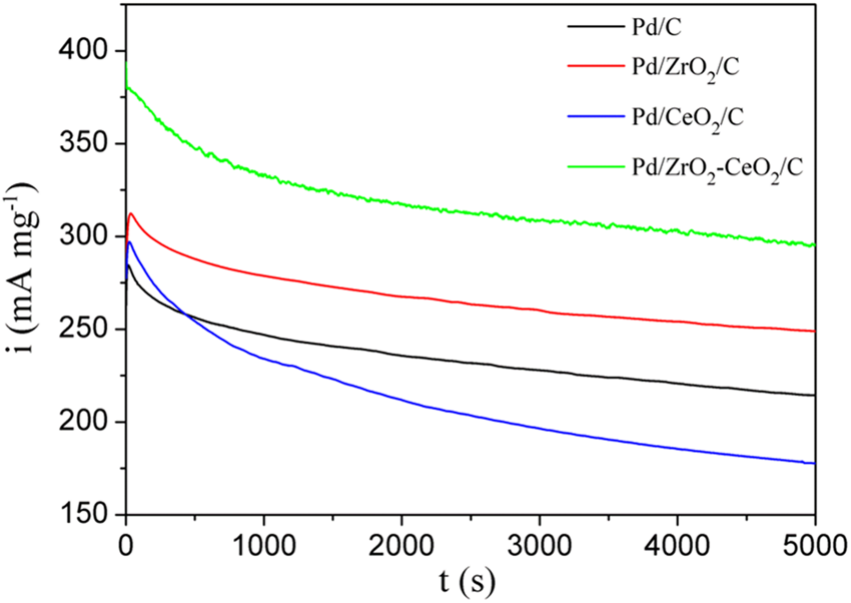
Chronoamperometric curves of different catalysts recorded at −0.25 V in 1 M KOH solution containing 1 M EG.

Moreover, the chronoamperometry curves of different catalysts were collected in 1 M KOH containing 1 M EG as shown in Fig. 6. It is clear that the Pd/ZrO_2_-CeO_2_/C catalyst has a highest initial anodic oxidation current density because of its best electrocatalytic activity in EG. Moreover, there are rapid decays of the current densities observed for all catalysts, due to the formation of CO-like intermediates during EG oxidation^13^. Then the current decreases slowly and reaches a pseudo-steady state after 4000 s. It is worth noting that the Pd/ZrO_2_-CeO_2_/C composite catalyst exhibits a highest steady state current density during the EG oxidation measurement, indicating its best stability among all catalysts in present work. The current density of Pd/ZrO_2_-CeO_2_/C catalyst is about 1.7 times as that of Pd/CeO_2_/C catalyst (295.5 vs. 177.8 mA. mg^−1^) after 5000 s.

**Figure 7:**
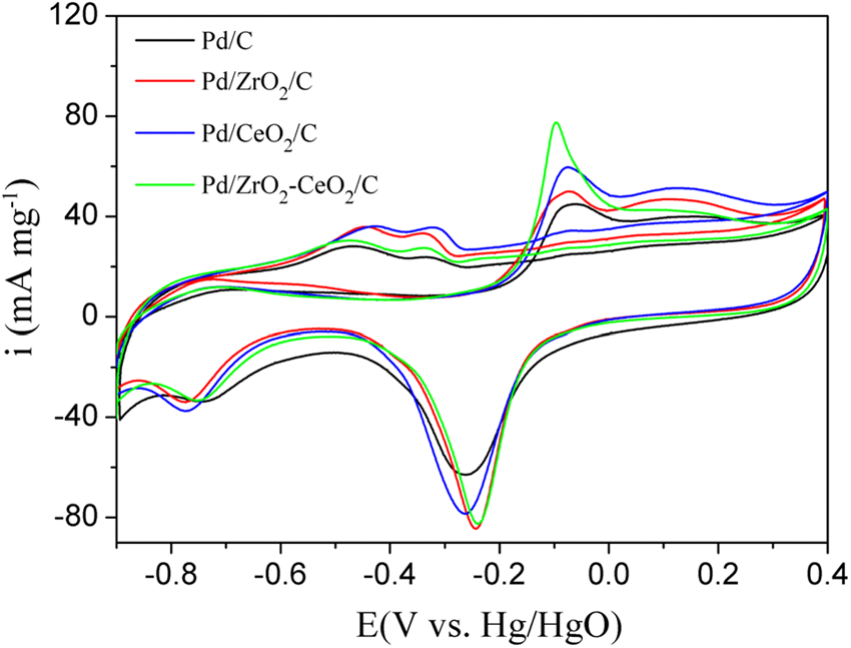
CO-stripping voltammograms of different catalysts in 1 M KOH solution at a scan rate of 50 mV s^−1^.

It is known that CO species are the main poisoning intermediate during the electro-oxidation process, thus a good catalyst for EG electro-oxidation should possess excellent CO electro-oxidizing ability. Figure 7 shows the CO-stripping voltammograms of different catalysts in 1 M KOH. It shows that the CO-striping onset potential of the Pd/ZrO_2_-CeO_2_/C composite catalyst is similar with all the catalysts studied, and only the peak areas are different, which is related to the electrochemical surface area.

Therefore, the electrochemical active surface area (EAS) is calculated by following equation^44, 45^

1 $${\rm{EAS}}={\rm{Q}}/{\rm{mC}}$$

where Q is the charge for CO desorption electro-oxidation, m is the amount of Pd loaded, and C (420 μC cm^−2^) is the charge needed for the adsorption of a CO monolayer. It is obvious that the EAS of the Pd/ZrO_2_-CeO_2_/C catalyst is larger than that of Pd/C (117.9 vs. 101.1 m^2^ g^−1^), which further supports the promoted CO-stripping of (Zr, Ce)O_2_ solid solution. To clarify the catalytic effect of ZrO_2_-CeO_2_, the specific activity of catalysts has been calculated by normalizing the cyclic voltammograms results (in Fig. 5) to EAS result. It is also worth noting that the specific activity obviously increases from 1.68 mA cm^−2^ for Pd/C to 3.14 mA cm^−2^ for Pd/ZrO_2_-CeO_2_/C catalyst. Therefore, the inclusion of (Zr, Ce)O_2_ solid solution enhances the stability as well as activity of Pd/C catalyst, which could be related to the strong interaction between Pd and (Zr, Ce)O_2_ solid solution and the weaker adsorption strength between the Pd surface and CO as discussed for XPS result in Fig. 3.

**Figure 8:**
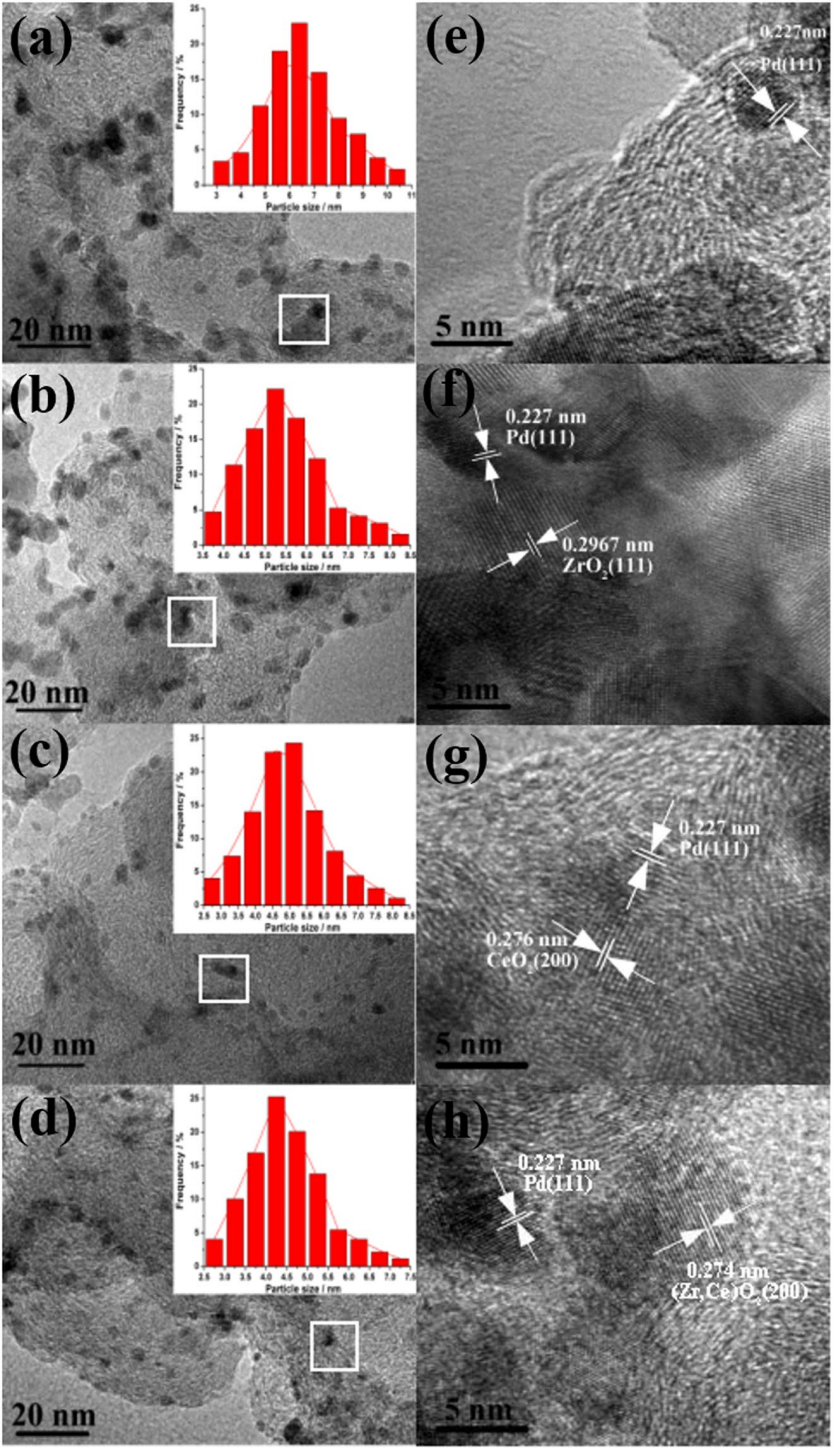
TEM images of different catalysts after 900 cycles in the 1 M KOH solution containing 1 M EG, for (**a**) Pd/C, (**b**) Pd/ZrO_2_/C, (**c**) Pd/CeO_2_/C and (**d**) Pd/ZrO_2_-CeO_2_/C. Insets show the corresponding size distribution in (**a**,**b**,**c**) and (**d**), respectively. (**e**,**f**,**g**) and (**h**) are the HRTEM images for (**a**,**b**,**c**) and (**d**), respectively.

**Figure 9:**
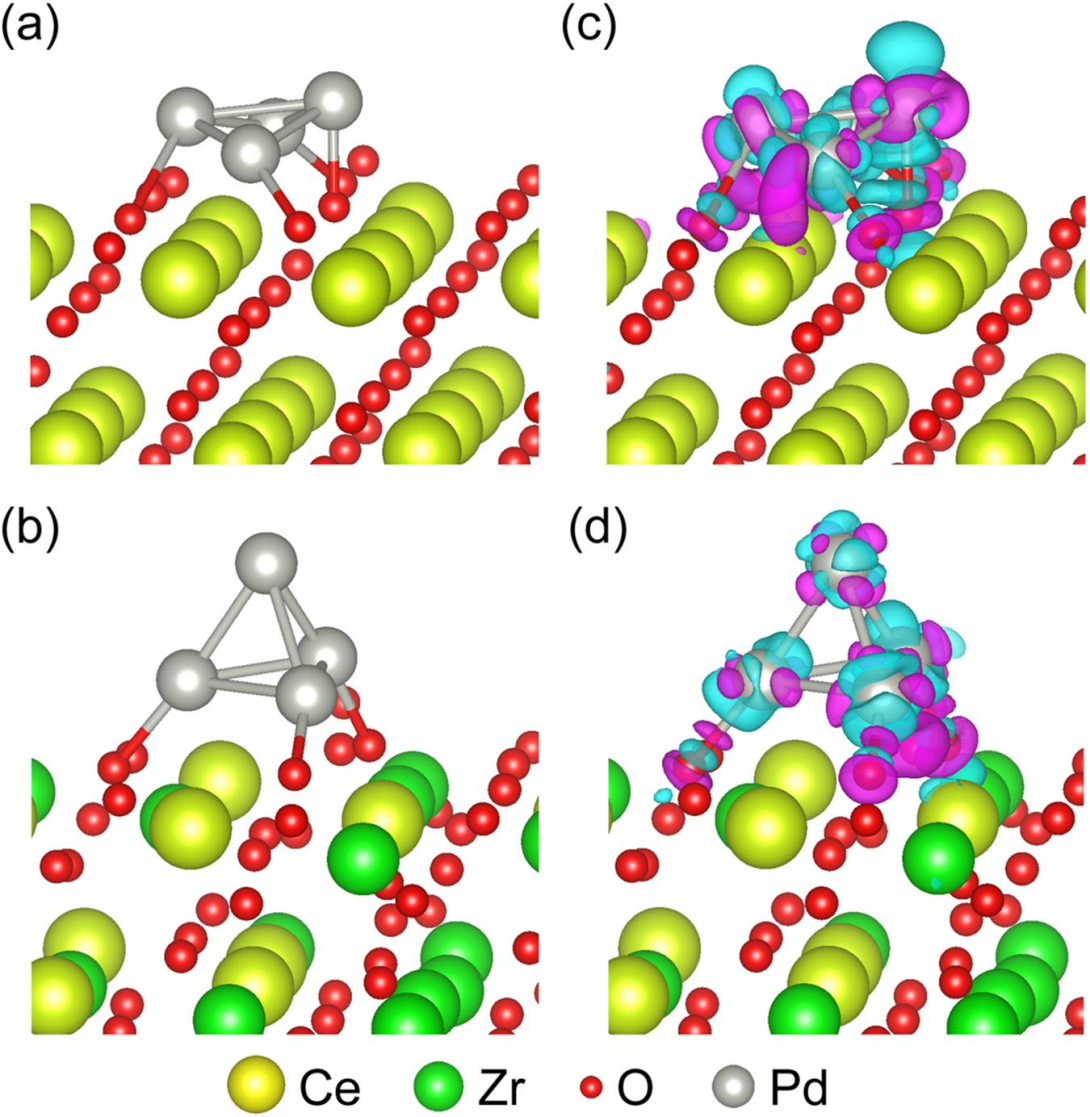
The relaxed (**a**) Pd_4_/CeO_2_ (111) and (**b**) Pd_4_/ZrO_2_-CeO_2_ (111) catalyst models used to study the CO absorption and desorption mechanism.

The TEM images and particle size distribution of different catalysts after 900 cycles in the 1 M KOH solution containing 1 M EG are displayed in Fig. 8. It shows that the average diameter of Pd nanoparticles after 900 cycles increases to 6.5, 5.5, 4.9 and 4.5 nm for Pd/C, Pd/ZrO_2_/C, Pd/CeO_2_/C and Pd/ZrO_2_-CeO_2_/C, respectively. The smaller increasement in diameter of Pd particles for Pd/ZrO_2_-CeO_2_/C has been obtained compared with that of catalysts before test as shown in Fig. 1, which indicates its better stability in the alkaline solution. This result can be attributed to protective decoration of Pd/C by (Zr, Ce)O_2_ solid solution which can prevent Pd from dissolution and agglomeration during cycling^46^. It is also observed that the d-spacing of 0.227 which belongs to the (111) plane of cubic Pd, from the HRTEM images of all catalysts in Fig. 9e–h, and the corresponding d-spacing of ZrO_2_, CeO_2_ and ZrO_2_-CeO_2_ have been measured and confirmed, which is similar as the TEM analyses of the catalysts before cycling in Fig. 1.

## Discussions

It is well established that the addition of oxides into Pd-based catalysts increases alcohol oxidation and the removal of adsorbed CO on the surface of catalysts. The higher catalytic activity of Pd/ZrO_2_-CeO_2_/C compared with that of Pd/CeO_2_/C in present work could be related to the addition of ZrO_2_ to CeO_2_. According to the bi-functional mechanism, the addition of ZrO_2_ probably more easily activates H_2_O to form oxygen-containing species (OH_ads_) at lower potential. Then the CO-like intermediate species could be reacted with these oxygen-containing species on the surface of Pd surface to release the active sites for further alcohol oxidation^30^. It has also been reported that the synergistic effect can be obtained if intimate mixing (solid solution) is achieved, then the electrochemical properties could be modified^47^. Therefore, the improved electrocatalytic activity and stability of Pd/ZrO_2_-CeO_2_/C composite in present work can be mainly attributed to the strong interaction between Pd and (Zr, Ce)O_2_ solid solution as well as the synergistic effect between CeO_2_ and ZrO_2_.

First principle calculations were performed to further unravel the physical origin of the improved electrocatalytic activity and stability of Pd/ZrO_2_-CeO_2_/C composite. Since the CeO_2_ (111) surface is more stable than CeO_2_(110) surface^48^, we have proposed a catalyst model with 3 × 3 CeO_2_ *p*(111) surface slab and a Pd 4-atom cluster, referred to as Pd_4_/CeO_2_ (111) hereafter. According to our previously analysis, we have obtained (Zr, Ce)O_2_ solid solution with CeO_2_ structure by replacing 50% Ce atoms with Zr atoms. Herein, the special quasi-random structure (SQS) approach^49^ has been introduced to model the (Zr, Ce)O_2_ solid solution. Corresponding catalyst model refers to as Pd_4_/ZrO_2_-CeO_2_ (111) hereafter. Figure 9 illustrates the relaxed structures of Pd_4_/CeO_2_ (111) and Pd_4_/ZrO_2_-CeO_2_ (111) catalyst models. Very interestingly, the Pd cluster behaviors is very different on top of the CeO_2_ and (Zr, Ce)O_2_ solid solution surface. As can be seen in Fig. 9a, for the Pd_4_/CeO_2_ (111) catalyst model, the Pd atoms spread on top of the CeO_2_ surface, forming a 2D plane structure. On the other hand, the four Pd atoms gather into a tilted tetrahedron on top of the surface of (Zr, Ce)O_2_ solid solution, forming a 3D stereoscopic cluster in Fig. 9b. The corresponding charge transfer between the Pd_4_ cluster and oxide surface *ρ*_chargetransfer_ illustrated in Fig. 8c and d are analyzed by:

2 $${\rho }_{{\rm{chargetransfer}}}={\rho }_{{{\rm{Pd}}}_{{\rm{4}}}}+{\rho }_{{\rm{Oxide}}}-{\rho }_{{\rm{catalyst}}}$$

where *ρ*_Pd4_, *ρ*_Oxide_ and *ρ*_catalyst_ are the self-consistent charge density of Pd_4_ clusters CeO_2_ (111) or ZrO_2_-CeO_2_ (111) surface, and Pd_4_/CeO_2_ (111) or Pd_4_/ZrO_2_-CeO_2_ (111) catalyst, respectively. As seen, the charge transfer between O and Pd atoms on the CeO_2_ (111) surface in Fig. 9c is stronger then on the ZrO_2_-CeO_2_ (111) surface, indicating that the chemical environment on the ZrO_2_-CeO_2_ (111) surface is more homogeneous. We performed the Bader charge^50^ analysis to quantify the transfer of charge density. The results show 0.592 *e* and 0.427 *e* net charge transferred from the Pd_4_ cluster to the CeO_2_ (111) and ZrO_2_-CeO_2_ (111) surface, respectively, indicating the 3D stereoscopic cluster is more stable than the 2D plane structure.

We assumed such structure difference leads to different anti-CO-poisoning properties. The required driving forces of CO desorption are evaluated by the following equation:

3 $${E}_{{\rm{desorption}}}={E}_{{\rm{CO}}}+{E}_{{\rm{catalyst}}}-{E}_{\mathrm{CO}/\mathrm{catalyst}}$$

where *E*_CO/catalyst_ shows the total energy of Pd_4_/CeO_2_ (111) or Pd_4_/ZrO_2_-CeO_2_ (111) catalyst with absorbed CO molecule, *E*_catalyst_ corresponds to the total energy of Pd_4_/CeO_2_ (111) or Pd_4_/ZrO_2_-CeO_2_ (111) catalyst, and *E*_CO_ is the total enegy of CO molecule. The corresponding CO desorption energies for the Pd_4_/CeO_2_ (111) and Pd_4_/ZrO_2_-CeO_2_ (111) catalyst are 2.759 eV and 1.836 eV. It is obviously that the desorption of the CO molecule from Pd_4_/ZrO_2_-CeO_2_ (111) catalyst is much easier than that from Pd_4_/CeO_2_ (111) catalyst. Hence, the Pd_4_/ZrO_2_-CeO_2_ (111) catalyst presents better anti-CO-poisoning properties, consistent with the best anti-CO-poisoning of the Pd/ZrO_2_-CeO_2_/C composite catalyst (in Fig. 7).

**Figure 10:**
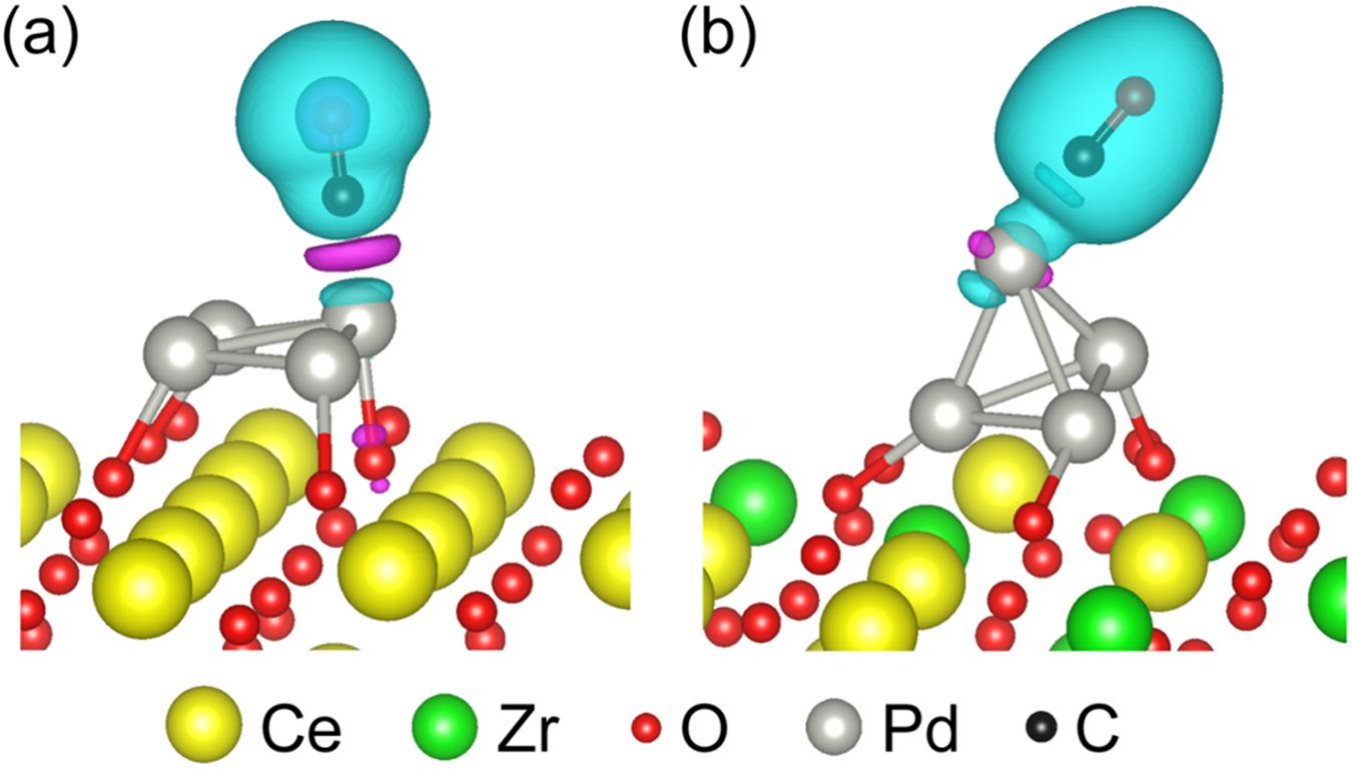
Plots of charge density difference (violet: charge depletion, cyan: charge accumulation) for CO absorbed on top of the (**a**) Pd_4_/CeO_2_ (111) and (**b**) Pd_4_/ZrO_2_-CeO_2_ (111) catalysts.

To further exploring the anti-CO-poisoning properties, the charge transfers from absorbed CO molecule to Pd cluster was studied by analyzing the charge density differences *ρ*_diff_, which is defined as:

4 $${\rho }_{{\rm{diff}}}={\rho }_{\mathrm{CO}/\mathrm{catalyst}}-({\rho }_{{\rm{catalyst}}}+{\rho }_{{\rm{CO}}})$$

where *ρ*_Co/catalyst_, *ρ*_catalyst_ and *ρ*_CO_ corresponding to the self-consistent charge density of Pd_4_/CeO_2_ (111) or Pd_4_/ZrO_2_-CeO_2_ (111) catalyst with absorbed CO molecule, Pd_4_/CeO_2_ (111) or Pd_4_/ZrO_2_-CeO_2_ (111) catalyst, and CO molecule, respectively. Figure 10 plots *ρ*_diff_ for CO molecule absorbed on Pd_4_/CeO_2_ (111) and Pd_4_/ZrO_2_-CeO_2_ (111) catalyst. The violet isosurfaces present the charge depletion *ρ*_diff_ < 0, while the cyan isosurfaces show the charge accumulation *ρ*_diff_ > 0. As seen in Fig. 10a, the absorption of CO on top of the Pd_4_/CeO_2_ (111) catalyst will lead to charge depletion in the middle of the CO molecule and the Pd 2D flat cluster, which contributes to strong Pd-C bonding. Meanwhile, for the case of CO absorbed on the Pd_4_/ZrO_2_-CeO_2_ (111) catalyst as shown in Fig. 10b, the charge accumulation between the CO molecule and the Pd 3D stereoscopic cluster increases the repulsive force between the C and Pd atom, resulting in a weaker Pd-C bonding. Combining with our previously analysis, the 3D stereoscopic Pd cluster on (Zr, Ce)O_2_ solid solution contributes to the good anti-CO-poisoning properties of Pd_4_/ZrO_2_-CeO_2_ (111) catalyst.

## Conclusions

In present work, the effect of (Zr, Ce)O_2_ solid solution on the structures and properties of Pd-based catalysts has been clearly demonstrated. The inclusion of (Zr, Ce)O_2_ solid solution reduces the particle size and increases the EAS of Pd nanoparticles, resulting in enhanced electrocatalytic activity of the composite catalyst for EG electro-oxidation in KOH solution. In addition, the strong interaction between Pd and (Zr, Ce)O_2_ solution decreases the Pd 3d electron density, which reduces the intermediate coverage on Pd surface and releases more active sites for EG electro-oxidation. The synergistic effect in (Zr, Ce)O_2_ also significantly increases the concentration of the OH_ads_ species on Pd surface and thus improves the stability and anti-CO-poisoning of Pd-based composite catalysts. Furthermore, the 3D stereoscopic Pd cluster on the surface of (Zr, Ce)O_2_ solid solution were explored based on first principle calculations, which leads to weaker Pd-C bonding and smaller CO desorption driving force. The findings on the good electrocatalytic properties of Pd/ZrO_2_-CeO_2_/C composite catalyst and the related mechanism will promote the development of stable catalyst for DAFCs application.

## Methods

### Sample preparation

26 mg of CeCl_3_·7H_2_O and 1 g urea (Sinopharm Chemical Reagent Co., Ltd, AR) were dissolved in 50 mL of deionized water. 84 mg Vulcan XC-72 carbon black was added into the solution, sonicated for 30 min and magnetic stirred for 1 h. The resulting solution was transferred into the oil bath at 90 °C for 3 h, washed with deionized water after cooling down naturally, and dried at 70 °C. The dried power was heated at 500 °C for 1 h in tube furnace under N_2_ atmosphere with a heating rate of 4 °C/min, then cooled down to get the support as CeO_2_/C. The ZrO_2_/C and ZrO_2_-CeO_2_/C were also prepared similarly. The molar ratio of ZrOCl_2_·8H_2_O (Sinopharm Chemical Reagent Co., Ltd, AR) to CeCl_3_·7H_2_O is 50:50 for preparing the ZrO_2_-CeO_2_/C. The mass ratio of the oxide to XC-72 carbon black is 1:7.

The catalysts were prepared through a borohydride reduction approach^43, 51^. 20 mg of PdCl_2_ (Shanghai Jiuyue Chemical Reagent Co., Ltd, AR) was sonicated in ultrapure water for 2 h. The prepared CeO_2_/C was sonicated in ultrapure water for 1 h. Two solutions were then mixed and stirred for 1 h, to obtain a solution with a nominal Pd-loading of 20 wt. %. The pH of this solution was adjusted to 9 using 1 M NaOH. NaBH_4_ was then added to the solution as a reduction agent, with a molar ratio of 1:8 for Pd and NaBH_4_. The resulting precipitate was filtered and washed with ultrapure water and ethanol, and then dried at 60 °C. The catalysts are designated as Pd/ZrO_2_/C, Pd/ZrO_2_-CeO_2_/C and Pd/CeO_2_/C, respectively. The Pd/C (Pd:C = 20:80 wt.%) was also prepared for comparison.

### Materials characterizations

The high resolution transmission electron microscopy (HRTEM) measurement was conducted in an Electron Microscope system (Tecnai G2 F20 S-TWIN) at 200 kV. Samples were prepared by transferring the catalyst suspension to a copper grid. The catalysts after 900 cycles in 1 M KOH containing 1 M EG were also subjected to TEM measurement.

The electronic structures of Zr 3d and Ce 3d, in different species before and after Pd-loading, were investigated by X-ray photoelectron spectroscopy (ESCALAB 250, Thermo Scientific, Inc.) with a monochromatic Al Kα source (10 mA, 15 kV), respectively. The electronic structure of Pd 3d in different catalysts was also measured by XPS. The spectra were fitted by the Gaussian-Lorentzian method, with a background subtraction by Shirley’s method^52^.

### Electrochemical measurements

3 mg of catalysts was added in 0.6 mL of Nafion solution (0.5 wt. %) and stirred for 30 min. 4 μL resulting ink was then transferred onto the electrode surface (glassy carbon disk, 3 mm in diameter), and dried at 70 °C for 10 min.

The electrochemical properties of catalysts were measured using an electrochemical workstation (CHI660D, Chenhua Inc., Shanghai, China) in a three-electrode system. The Pt plate (1 cm^2^) was served as the counter electrode, a Hg/HgO electrode for the reference electrode, and the glassy carbon disk electrode for the working electrode. All measurements were conducted in the water bath of 25 ± 1 °C. The measured Pd loading in the catalysts was 0.057 mg cm^−2^, by an inductively coupled plasma equipped with with atomic emission spectroscopy (ICP-AES).

The chronoamperometry was performed at a potential of −0.25 V (1 M KOH containing 1 M EG). In the electrochemical CO-stripping measurements, CO was bubbled into the solution for 15 min with a fixed the catalyst potential (0 V vs. Hg/HgO). The residual CO in the solution was removed by N_2_ (99.9%). The cyclic voltammograms (CVs) were conducted in the potential ranging from −0.9 V to 0.4 V vs. Hg/HgO with a sweep rate of 50 mV s^−1^ (1 M KOH and 1 M KOH containing 1 M EG).

### First principle calculations

Our first principle calculations based on the density functional theory, were performed using the Vienna ab initio simulation package (VASP)^53^ in conjunction with the projector augmented wave (PAW) generalized gradient approximations (GGA)^54^ of Perdew-Burke-Ernzerhof (PBE)^55^ pseudopotentials. The valence electron configurations for C, O, Zr and Ce were 2*s* ^2^2*p* ^2^, 2*s* ^2^2*p* ^4^, 4*s* ^2^4*p* ^6^5*s* ^2^4*d* ^2^ and 5*s* ^2^5*p* ^6^6*s* ^2^4*f* ^1^5*d* ^1^, respectively. The geometry convergence was achieved with the Γ symmetry K-point using the Gaussing smearing (σ = 0.02 eV) with the cut-off energy of 500 eV. The CeO_2_ *p*(111) surface slab is modeled by a (3 × 3) unit cell. The relaxation convergence for ions and electrons were 1 × 10^−5^ and 1 × 10^−6^ eV, respectively. The crystal structures and polyhedrons were visualized using the VESTA tool^56^.

## Electronic supplementary material

Supplementary Information
