# Supplementary material for: Improving the electrocatalytic properties of Pd-based catalyst for direct alcohol fuel cells: effect of solid solution
Source: Sci Rep. 2017 Jul 7;7:4907. doi: 10.1038/s41598-017-05323-y (PMC5501847; doi:10.1038/s41598-017-05323-y)
Supplement: Supplementary file 1 — Supplementary Information [file 41598_2017_5323_MOESM1_ESM.doc]

**Supporting Information**

**Improving the electrocatalytic properties** **of Pd-based catalyst for direct alcohol fuel cells: effect of solid solution**

Cuilian Wen^a^, Ying Wei^a^, Dian Tang^a^, Baisheng Sa^a^,*, Teng Zhang^a^,*,

and Changxin Chen^a,b^,*

^a^ College of Materials Science and Engineering, Fuzhou University, Fuzhou, Fujian 350116, P. R. China

^b^ National Key Laboratory of Science and Technology on Micro/Nano Fabrication, Key Laboratory for Thin Film and Microfabrication of the Ministry of Education, Department of Micro/Nano Electronics, School of Electronic Information and Electrical Engineering, Shanghai Jiao Tong University, Shanghai 200240, China

**Experimental**

The crystalline structure of the species before or after Pd-loading was measured using an X-ray diffractometer (XRD, Rigaku D/max-IIIC), with a copper Kα source (λ = 1.5406 Å) .

**Results and Discussion**

*XRD results of the samples:*

The XRD patterns of different catalyst supports are given in Fig. S1a. It is worth noting that the XRD patterns of ZrO_2_-CeO_2_/C are very similar to that of CeO_2_/C, except that the Bragg angles shift positively compared with that of CeO_2_/C. Considering the smaller size of Zr^4+^ ions compared with that of Ce^4+^ ion (0.72 vs. 0.87 Å)[^1^](#_ENREF_1), the positive shift of the Bragg angles and the decrease in d-spacing for ZrO_2_-CeO_2_/C indicate that the formation of the (Zr, Ce)O_2_ solid solution with the addition of ZrO_2_[^2^](#_ENREF_2).

In addition, the characteristic diffraction peaks of Pd are clearly recognized from Fig. S1b. The Pd (111) peak was introduced to calculate the particle size of Pd based on the Sherrer’s equation. The average Pd particle sizes are 4.6, 4.0, 4.4 and 3.8 nm for the Pd/C, Pd/CeO_2_/C, Pd/ZrO_2_/C and Pd/ZrO_2_-CeO_2_/C catalysts respectively.

**Fig. S1a**


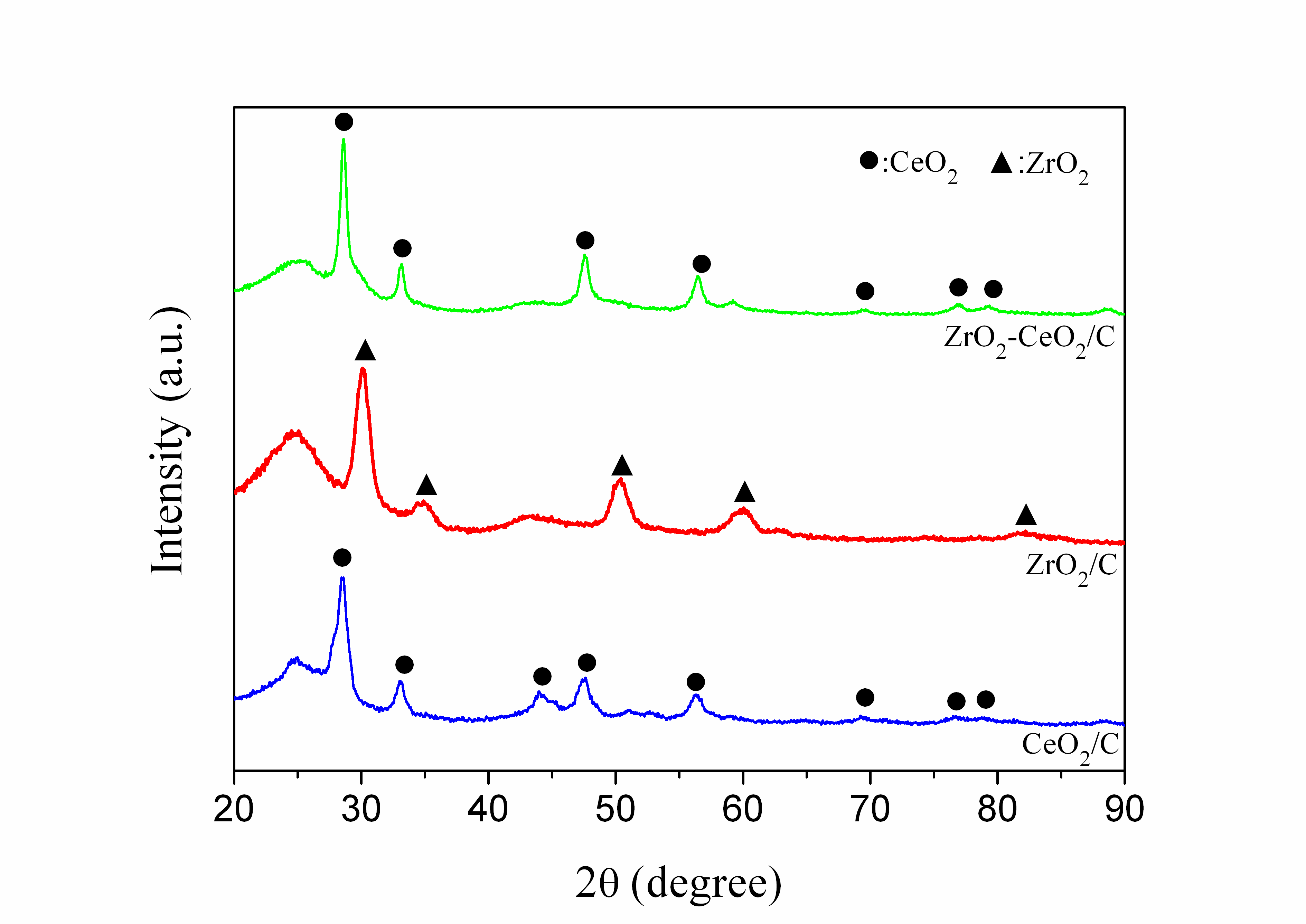


**Fig. S1b**


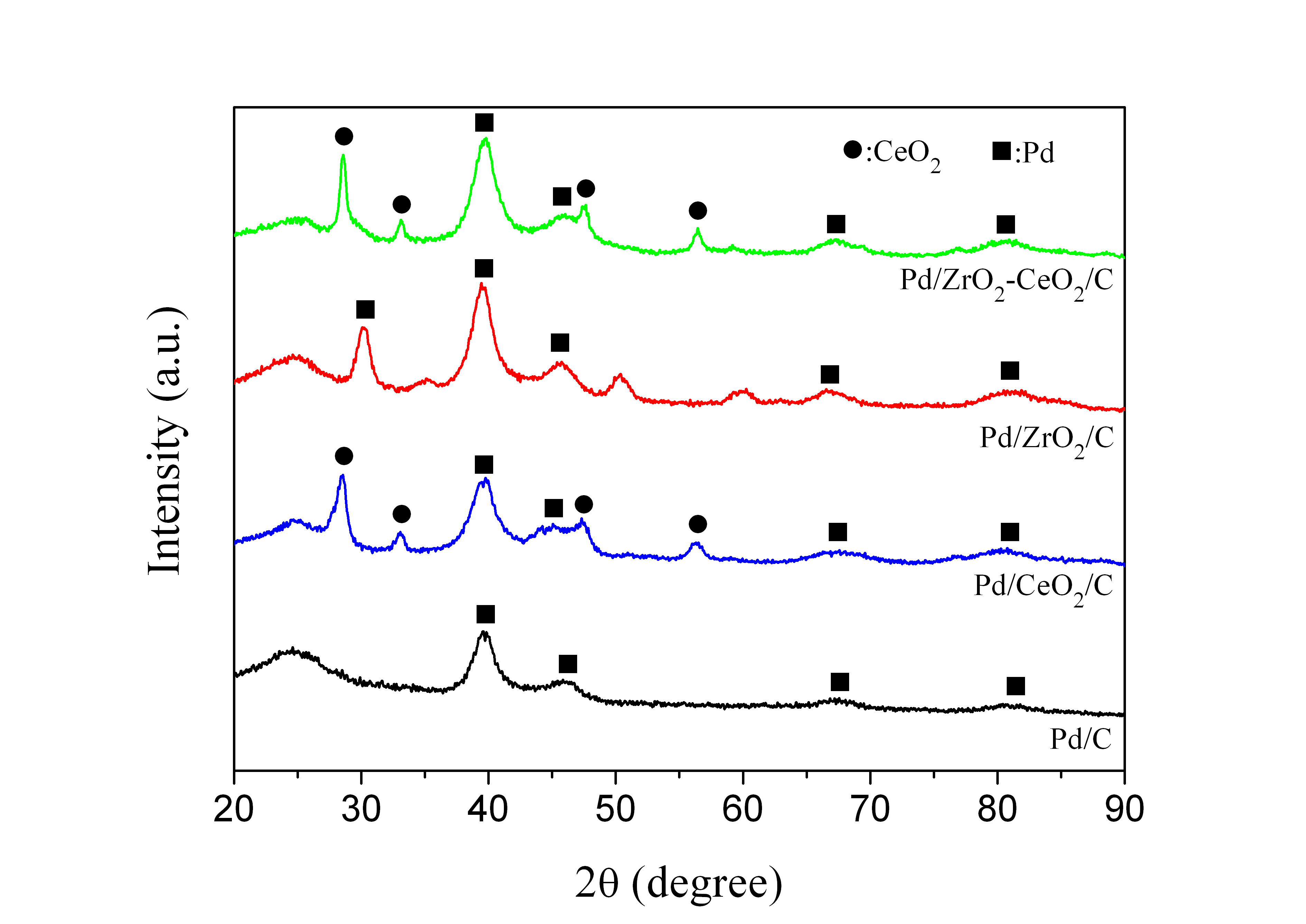


Fig. S1. XRD patterns of (a) different catalyst supports and (b) different catalysts.

**References**

1. Shannon, R. D. Revised effective ionic radii and systematic studies of interatomic distances in halides and chalcogenides. *Acta Crystallographica Section A* **32**, 751-767 (1976).

2. Chen, L. F. *et al.* Surfactant-controlled synthesis of Pd/Ce0.6Zr0.4O2 catalyst for NO reduction by CO with excess oxygen. *Applied Surface Science* **243**, 319-328 (2005).

3. Cui, Q. *et al.* Based on a new support for synthesis of highly efficient palladium/hydroxyapatite catalyst for ethanol electrooxidation. *Electrochimica Acta* **132**, 31-36 (2014).

4. Zhang, M. *et al.* In situ synthesis of palladium nanoparticle on functionalized graphene sheets at improved performance for ethanol oxidation in alkaline media. *Electrochimica Acta* **111**, 855-861 (2013).
